# Definitions, End Points, and Clinical Trial Designs for Bladder Cancer: Recommendations From the Society for Immunotherapy of Cancer and the International Bladder Cancer Group

**DOI:** 10.1200/JCO.23.00307

**Published:** 2023-10-04

**Authors:** Ashish M. Kamat, Andrea B. Apolo, Marek Babjuk, Trinity J. Bivalacqua, Peter C. Black, Roger Buckley, Matthew T. Campbell, Eva Compérat, Jason A. Efstathiou, Petros Grivas, Shilpa Gupta, Neil J. Kurtz, Donald Lamm, Seth P. Lerner, Roger Li, David J. McConkey, Joan Palou Redorta, Thomas Powles, Sarah P. Psutka, Neal Shore, Gary D. Steinberg, Richard Sylvester, J. Alfred Witjes, Matthew D. Galsky

**Affiliations:** ^1^ Department of Urology, The University of Texas MD Anderson Cancer Center, Houston, TX; ^2^ Center for Cancer Research, National Cancer Institute, NIH, Bethesda, MD; ^3^ Department of Urology, Teaching Hospital Motol, 2^nd^ Faculty of Medicine, Charles University, Prague, Czech Republic; ^4^ Division of Urology, Department of Surgery, University of Pennsylvania, Philadelphia, PA; ^5^ Department of Urologic Sciences, University of British Columbia, Vancouver, British Columbia, Canada; ^6^ Department of Urology, North York General Hospital, Toronto, Ontario, Canada; ^7^ Department of Genitourinary Medical Oncology, Division of Cancer Medicine, The University of Texas MD Anderson Cancer Center, Houston, TX; ^8^ Department of Pathology, Medical University of Vienna, Vienna, Austria; ^9^ Department of Radiation Oncology, Massachusetts General Hospital, Harvard Medical School, Boston, MA; ^10^ Department of Medicine, Division of Oncology, University of Washington; Clinical Research Division, Fred Hutchinson Cancer Center, Seattle, WA; ^11^ Department of Hematology and Medical Oncology, Cleveland Clinic Taussig Cancer Institute, Cleveland, OH; ^12^ Patient Advocate, Bladder Cancer Advocacy Network (BCAN), Bethesda, MD; ^13^ BCG Oncology, Phoenix, AZ; ^14^ Scott Department of Urology, Dan L Duncan Cancer Center, Baylor College of Medicine, Houston, TX; ^15^ Department of Genitourinary Oncology, H Lee Moffitt Cancer Center, Tampa, FL; ^16^ Johns Hopkins Greenberg Bladder Cancer Institute, Johns Hopkins University, Baltimore, MD; ^17^ Department of Urology, Fundació Puigvert, Universitat Autònoma de Barcelona, Barcelona, Spain; ^18^ Queen Mary University of London, London, United Kingdom; ^19^ Department of Urology, University of Washington, Fred Hutchinson Cancer Center, Seattle, WA; ^20^ Carolina Urologic Research Center, Myrtle Beach, SC; ^21^ Department of Urology, Rush University Medical Center, Chicago, IL; ^22^ EAU NMIBC Guidelines Panel, Arnhem, the Netherlands; ^23^ Radboud University Medical Center, Nijmegen, the Netherlands; ^24^ Icahn School of Medicine at Mount Sinai, Tisch Cancer Institute, New York, NY

## Abstract

**PURPOSE:**

There is a significant unmet need for new and efficacious therapies in urothelial cancer (UC). To provide recommendations on appropriate clinical trial designs across disease settings in UC, the Society for Immunotherapy of Cancer (SITC) and the International Bladder Cancer Group (IBCG) convened a multidisciplinary, international consensus panel.

**METHODS:**

Through open communication and scientific debate in small- and whole-group settings, surveying, and responses to clinical questionnaires, the consensus panel developed recommendations on optimal definitions of the disease state, end points, trial design, evaluations, sample size calculations, and pathology considerations for definitive studies in low- and intermediate-risk nonmuscle-invasive bladder cancer (NMIBC), high-risk NMIBC, muscle-invasive bladder cancer in the neoadjuvant and adjuvant settings, and metastatic UC. The expert panel also solicited input on the recommendations through presentations and public discussion during an open session at the 2021 Bladder Cancer Advocacy Network (BCAN) Think Tank (held virtually).

**RESULTS:**

The consensus panel developed a set of stage-specific bladder cancer clinical trial design recommendations, which are summarized in the table that accompanies this text.

**CONCLUSION:**

These recommendations developed by the SITC-IBCG Bladder Cancer Clinical Trial Design consensus panel will encourage uniformity among studies and facilitate drug development in this disease.

## INTRODUCTION

A significant unmet need exists for new and effective treatments for urothelial cancers (UCs). To identify optimal treatment options and improve outcomes for patients, trial design is of utmost importance—including rationally selected end points, eligibility criteria, evaluations, statistical analyses, and correlative studies. Standardizing trial design and end points benefits the field by creating more comparable and robust data sets for meta-analyses.

Following up on prior efforts of the International Bladder Cancer Group (IBCG)^1^ and the Society for Immunotherapy of Cancer (SITC),^2^ we present a consensus statement to provide guidance to investigators for rigorous (late phase) clinical trial design, exposing patients to agents with a high likelihood of antitumor efficacy yielding data that best advance the field.

## METHODS

See the Data Supplement (online only) for a discussion of the following:

• Consensus panel composition
• Conflict of interest management
• Recommendation development

## RESULTS

See the Data Supplement for a discussion of the following:

• Critical value for effect size and sample size considerations
• Relevant histologic subtypes and variants

Generally, clinical trials for novel agents for the treatment of bladder cancer should be designed to demonstrate superiority to the current standards—noninferiority trial designs may be appropriate in some settings but are complicated because the margin for unacceptable loss in efficacy is subjective and demonstrating differences at small margins requires large numbers of patients. A summary of the recommendations for eligibility, design, end points, comparators, assessments, and sample size considerations across bladder cancer disease states is provided in Table 1.

**TABLE 1.**
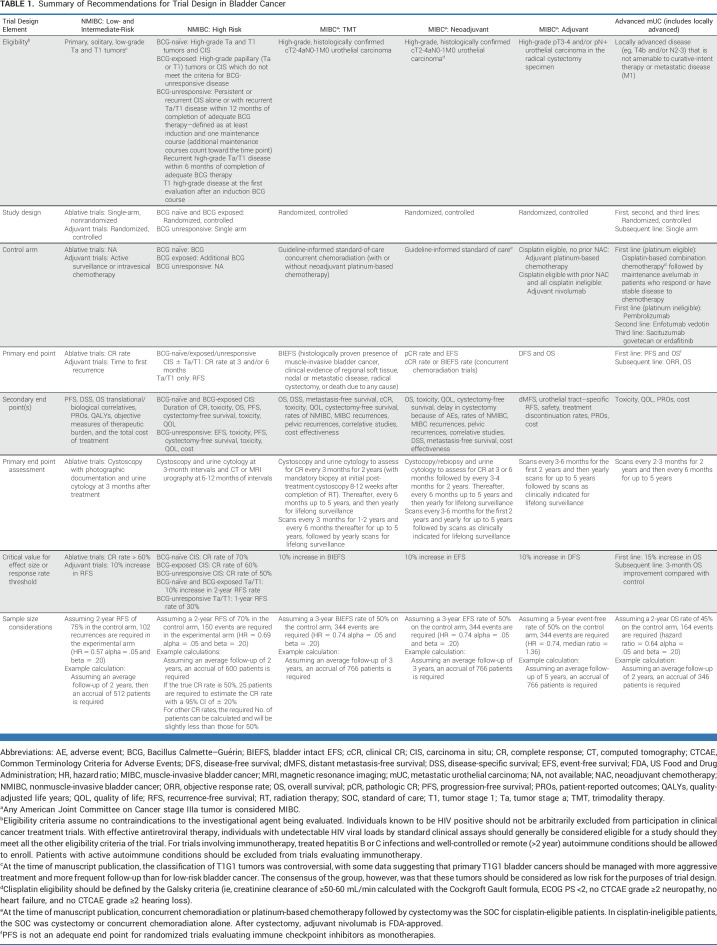
Summary of Recommendations for Trial Design in Bladder Cancer

Additional considerations:

- All trials of UC should include objective quality-of-life (QOL) end points,^3^ with attention to the overall cost of treatment.^4^
- Progression should always be counted as a recurrence event.
- UC frequently displays different histologic features, with several distinct histologic subtypes and patterns of differentiation.^5^

## NON-MUSCLE INVASIVE BLADDER CANCER

See the Data Supplement for a discussion of the following:

• Exploratory analyses
• Treatment failure definition
• Recurrence definition
• Additional considerations

## NMIBC: LOW- AND INTERMEDIATE-RISK

See the Data Supplement for a discussion of the following:

• Disease state definition
• Statistical and pathology considerations

### Research Hypothesis

The objectives for clinical trials of low- and intermediate-risk non-muscle invasive bladder cancer (NMIBC) are to (1) test the antitumor activity and (2) test the ability of the treatments to prevent disease recurrence or progression.

### Study Objectives

Two types of trial design should be permitted: ablative (conceptually similar to neoadjuvant) and adjuvant therapy trials.

### Study Objectives for Ablative Trials

In ablative trials, the objective is to treat existing tumors by means other than surgical resection (the current standard of care [SOC]). Treatment can be initiated after diagnosis with the tumor(s) left in situ; the baseline tumor may be a marker lesion (approximately 0.5-1 cm in diameter) left behind after all other lesions are resected. These trials provide direct evidence for ablative activity as measured by complete response (CR) rate (in phase II/III studies) of the investigational agent at an early time point.

CR rate, the primary end point for phase II studies, is defined by absence of disease at the treated tumor sites at 3 months as seen on cystoscopy and negative urine cytology, with the option of surgical sampling of the post-treatment scar for histological evaluation. Historical analyses of marker lesion studies demonstrated that biopsy at the site of the marker lesion scar rarely revealed histological presence of tumor (3 of 110 cases).^6^ However, the 2022 randomized phase II CALIBER feasibility trial of chemoablation with mitomycin-C (MMC) compared with surgical management in low-risk NMIBC reported disease confirmed on biopsy in 6 of 26 patients with no visible disease after chemoablation.^7^ Although 3-month CR rate does not necessarily correspond to durable clinical benefit, historical marker lesion trials observed CR rates of roughly 60% with intravesical chemotherapy.^8^

Secondary end points should include recurrence-free survival (RFS) in complete responders and progression-free survival (PFS) as defined by the time to progression to high-grade (HG)/stage disease. Additionally, an event that precludes further assessment of recurrence/progression (ie, nonbladder cancer–related death or radical cystectomy) will be a competing risk and necessitates that time to event curves are estimated using cumulative incidence functions.

### Study Objectives for Adjuvant Therapy Trials

The primary end point in adjuvant therapy trials of drugs used after transurethral resection of bladder tumor (TURBT) should be time to first recurrence compared by the log-rank test. Recurrence of NMIBC or disease progression constitutes recurrence events. Secondary end points may include PFS, disease-specific survival, overall survival (OS), and safety.

### Study Design

Ablative and adjuvant therapy differ in the need for random assignment and other characteristics.

### Study Design for Ablative Trials

Ablative therapy trials should be conducted using phase I, single-armed designs or randomized between different agents or different doses of the same agent to assess safety. To assess activity, a phase II/III design should be used. Dose escalation is allowed. Once the recommended phase II dose is determined, the investigational agent should be evaluated in expanded phase II studies to further assess CR and other end points. Although single-arm studies are permitted in phase II, assessment of activity end points is more meaningful in randomized controlled trials comparing the ablative activity of the agent with TURBT and the ability of the investigational agent to prevent tumor recurrence.

### Study Design for Adjuvant Therapy Trials

Adjuvant therapy trials should be randomized.^9^ Until the current Bacillus Calmette–Guérin (BCG) shortage is resolved, there is consensus that intravesical BCG should be reserved for patients with high-risk NMIBC.^10-12^ Patients enrolled into the comparator arm of adjuvant therapy trials for low-risk NMIBC should either be actively surveilled or treated with standardized intravesical chemotherapy.^9^ For intermediate-risk patients, the SOC comparator depends on the number of risk factors, including multifocal tumors, early recurrence (<1 year), frequent recurrences (>1 per year), large tumors (≥3 cm), and failure of prior intravesical treatment. Per IBCG consensus recommendations, one dose of postoperative intravesical chemotherapy is appropriate for patients with no risk factors. Additional adjuvant induction intravesical chemotherapy (or BCG if prior chemotherapy has been used) should be offered to patients with ≥1 risk factor(s).

Complete transurethral resection should be performed before study enrollment. Single-course postoperative intravesical chemotherapy is allowed, but not mandatory. Specifying which patients received postoperative treatment is essential. Analysis should be stratified by postoperative intravesical chemotherapy to account for this as a confounding factor and by institution in multicenter studies.

### Evaluation and Follow-Up

Baseline evaluation for NMIBC studies should include bladder cancer history detailing the date of initial diagnosis, grade, stage, multiplicity, tumor size, number of prior recurrences, previous treatment history, and detailed cystoscopic findings including tumor appearance with accompanying cystoscopic photographs, tumor location, multiplicity, and estimated size. For assessment, if advanced cystoscopic techniques such as blue light are used, they should be used consistently at baseline and on-treatment, and their use should be documented. Urine cytology may be used to rule out occult HG disease. Evaluation of urinary biomarkers may help; however, these assays lack validation in large cohorts, and the potential for false positives should be taken into account.^13^ Additionally, contrast-enhanced cross-sectional imaging obtained to rule out concomitant upper tract urothelial carcinoma, lymphadenopathy, and associated metastatic disease should be noted.

After treatment completion in ablative trials, assessment of the index tumor/scar using cystoscopy and urine cytology is required at 3 months to document therapeutic activity while biopsy of the index tumor/scar is optional for translational/biologic correlative studies. For ablative therapy trials, if residual tumor is present after therapy, SOC treatment should be instituted. Maintenance therapy using the investigational agent should be allowed in patients without recurrence. Alternatively, if any residual tumor can be completely resected or if no residual tumor is found, the patient may be enrolled in an adjuvant therapy trial using the same investigational agent depending on its mechanism of action. Surveillance cystoscopy evaluation and urine cytology should be conducted at 3-4 months of intervals for the first year and every 6 months thereafter for 2 years.^14,15^ These time points are also convenient for urine collection and analysis.

## NMIBC: HIGH RISK

See the Data Supplement for a discussion of the following:

• Disease state definition
• Statistical and pathology considerations

### Research Hypothesis

Further augmentation of the antitumor immune response for patients with high-risk NMIBC with the investigational agent in addition to BCG, new strain of BCG, or alternative to BCG (in BCG-naïve and BCG-exposed NMIBC) or after BCG (in BCG-unresponsive NMIBC) will more effectively eradicate cancer cells compared with treatment with BCG alone (in BCG-naïve and BCG-exposed NMIBC) or historic controls. In BCG-unresponsive carcinoma in situ (CIS), a randomized trial is recommended for papillary disease, thereby inducing a durable CR for CIS or CR or prolonging event-free survival (EFS) for Ta and T1 tumors.

### Study Objectives

In scenarios where patients with CIS (with or without papillary disease) and papillary-only disease are evaluated separately, CR rate at 6 months and RFS, respectively, are appropriate primary end points. RFS in this setting includes death as an event and not a competing risk, distinct from low- and intermediate-risk NMIBC. Because CIS is not expected to be fully resected, treatment response is the primary efficacy measure. In single-arm studies, a primary end point of CR rate at 6 months^16^ and a secondary end point of duration of CR (defined as the time from first response to disease progression or death) are recommended in trials evaluating novel agents in the BCG-unresponsive CIS setting. In patients with papillary-only disease, tumors are required to be fully resected before study entry. Therefore, treatment is administered in the adjuvant setting to prevent recurrence. Given the inherent difficulty in measuring benefit in this population, BCG-naïve papillary-only disease trials require random assignment and a control arm to measure benefit of novel therapeutics.

### Study Design

Because untreated high-risk NMIBC has a high likelihood of disease progression, comparison with a placebo would be unethical. For untreated BCG-naïve high-risk NMIBC, the SITC-IBCG panel recommends randomly assigning patients to the investigational agent versus a control protocol including standard BCG induction followed by once per week for 3 weeks (ie, Southwest Oncology Group [SWOG] schedule) maintenance treatments administered at 3 months, 6 months, and then every 6 months for at least 1 year.^17^

In the BCG-exposed setting, comparison of experimental therapies with a control arm receiving a placebo in combination with BCG if the treatment arm includes BCG plus a study drug is recommended.^18^

Importantly, the ongoing BCG shortage^10-12^ may affect the feasibility of following this optimal study design, and therefore, one-third dose BCG or an alternate SOC agent may be an acceptable comparator arm.

In the BCG-unresponsive setting, radical cystectomy is considered the most effective definitive treatment,^16^ yet many patients are reluctant to consider this option or are not surgical candidates.^19^ Random assignment in this setting may be less acceptable to patients/providers, and single-arm trial designs have been permitted by the US Food and Drug Administration (FDA).^20^ For populations with CIS, CR rate and duration are clinically meaningful end points. Per FDA guidance, CR rate and duration should be benchmarked against historical agents or modern options (eg, valrubicin, pembrolizumab, gemcitabine and docetaxel, or nadofaragene firadenovec).^21^ For patients with BCG-unresponsive CIS, who have recurrence of CIS at 3 months, one additional course of treatment is allowed as historically, 60% of patients with persistent CIS after initial induction BCG will convert to CR after a reinduction or first BCG maintenance course.^17,22,23^ Patients with papillary-only disease in the BCG-unresponsive setting require random assignment to observation or an accepted community standard (eg, intravesical gemcitabine and docetaxel).^24,25^ Stratification criteria should include key prognostic factors.

### Study Population

Patients with prostatic urethral involvement have been excluded in most trials; however, the SITC-IBCG panel recommends that these patients be included but stratified for random assignment. While regulatory agencies prefer separating patients with CIS and papillary-only disease, the SITC-IBCG panel recommends randomized studies with pooling of mixed populations of patients with CIS (with or without papillary disease) and papillary-only disease where a composite end point of EFS can be used to evaluate the treatment effect of a novel therapy. Histologic subtypes should be allowed, and a stratification factor for the presence of >50% histologic variation should be considered. Events should include persistence of CIS at 6 months, development of any HG Ta/CIS/T1 within the bladder or remaining urinary tract, or development of muscle-invasive/advanced bladder cancer or death due to any cause. As most of the events will be NMIBC, especially in the BCG-naïve setting, permitting treatment beyond persistence/recurrence in the absence of stage progression should be considered. In the era of checkpoint inhibitors, delayed and atypical responses have been observed in advanced disease, albeit infrequently.^26^ Treatment and response kinetics unique to certain therapies should be considered on the timing of treatment failure assessment.

### Evaluation and Follow-Up

Regardless of the primary end point, cystoscopy and urine cytology at 3-month intervals and computed tomography (CT) or magnetic resonance imaging (MRI) urography at 6-12 months of intervals should be used for efficacy evaluation. If used, advanced cystoscopy techniques such as blue light cystoscopy^27^ should be performed and documented consistently at baseline and on-treatment. Random or bladder-mapping biopsies at fixed time points are not performed as usual SOC in either the BCG-naïve or unresponsive setting^28^ and were not required in the 2018 FDA guidance for BCG unresponsive disease.^29^ This panel, however, recommends random 12-month bladder biopsies as an option for high-risk NMIBC trials.

Given the natural history of high-risk NMIBC, the majority of recurrence or progression events will occur within the first 2 years from start of treatment,^30^ with fewer events in subsequent years. Thus, an adequate study duration should be at least 2 years, which will also permit adequate evaluation of key response end points, with longer follow-up for survival end points.

## MUSCLE-INVASIVE BLADDER CANCER: NEOADJUVANT

See the Data Supplement for a discussion of the following:

• Disease state definition
• Baseline staging and stratification
• Statistical and pathology considerations

### Research Hypothesis

Systemic treatment before cystectomy may be better tolerated or more feasible and offers earlier treatment of micrometastatic disease and measurement of pathological response of the primary tumor as an intermediate measure of treatment benefit. Neoadjuvant cisplatin-based chemotherapy has demonstrated improved survival in patients with muscle-invasive bladder cancer (MIBC).^31,32^ However, rates of metastatic recurrence remain relatively high, and a large subset of patients are ineligible to receive cisplatin-based chemotherapy.

### Study Objectives

The primary objective of neoadjuvant therapy is to eradicate micrometastases and decrease primary tumor burden before consolidative local therapy. Pathologic CR (pCR) after chemotherapy (ypT0N0) is associated with improved OS.^31^ Patients who achieve ypTcisN0 similarly experience excellent outcomes.^33,34^ However, using pCR as a trial-level surrogate for OS has not been established in MIBC, particularly in trials evaluating neoadjuvant immunotherapy. The SITC-IBCG panel recommends that pCR as a primary end point in a phase III trial be accompanied by a coprimary end point, such as EFS. Trials of concurrent chemoradiation may use clinical CR (cCR) as an intermediate end point, although bladder intact EFS (BIEFS) is considered the primary end point of choice by this panel.

The treatment-associated adverse event (AE) rate should be a secondary end point, including the percentage of patients precluded from cystectomy or completion of planned chemoradiation because of toxicity, rates of immune-related AEs, delay to cystectomy because of AEs, and rates of postcystectomy or postradiation complications. Other important secondary end points recommended by this panel include overall survival, metastasis-free survival, cystectomy-free survival, preservation of bladder function, and NMIBC or MIBC recurrences for bladder-preserving strategies.

### Study Design

Neoadjuvant studies of MIBC should be randomized phase III trials comparing current SOC with the experimental therapy. Generally, neoadjuvant therapy is followed by radical cystectomy with pelvic lymph node dissection. However, trimodality therapy (TMT), incorporating maximal TURBT followed by chemoradiation, is increasingly recognized as SOC for MIBC.^35^ Trials may allow for both radical cystectomy and bladder-preserving strategies with radiation-based therapy, as was done in the BA06 30894 trial.^32^

Patients with MIBC are classified into four broad groups on the basis of cisplatin and cystectomy eligibility, with implications for comparator arm design. For cisplatin-eligible patients, either concurrent chemoradiation or cisplatin-based neoadjuvant chemotherapy (NAC) followed by cystectomy should be used as the SOC comparator. It is standard to give four cycles of NAC (cycle length of approximately 3 weeks). For cisplatin-ineligible patients, cystectomy alone followed by adjuvant anti–PD-1 or concurrent chemoradiation should be used as the SOC comparator. Current phase III trials include studies of patients who are cisplatin-eligible and cystectomy-eligible, cisplatin-ineligible and cystectomy-eligible, and those enrolling to bladder-sparing protocols including chemoradiation or intravesical chemotherapy combined with systemic checkpoint blockade.^36^

Approximately half of patients with MIBC are not eligible to receive cisplatin.^37^ The current SOC at the time of manuscript publication for these patients was immediate radical cystectomy, followed by adjuvant nivolumab or TMT without neoadjuvant therapy.

### Study Population

Cisplatin eligibility can be delineated using the Galsky criteria (with flexibility as related to the AE profile of the experimental regimen being investigated), including a creatinine clearance of ≥50-60 mL/min (Cockcroft Gault); Eastern Cooperative Oncology Group performance status (ECOG PS) <2; and an absence of ≥grade 2 neuropathy, NYHA III/IV heart failure, or ≥grade 2-3 hearing loss.^38^ Some investigators support using alternative methods of calculating renal function (eg, Modification of Diet in Renal Disease formula, CKD Epidemiology Collaboration equation adjusting for body surface area) and changing the threshold to include patients with a GFR ≥50 mL/min.^37^ Immune checkpoint inhibitor (ICI) therapy adds limited additional risk to patients with well-controlled HIV infection, treated hepatitis B or C infections, and well-controlled or remote autoimmune conditions, so these patients should not be excluded from trials.^39,40^ For radiotherapy eligibility, patients must not have had prior pelvic radiation and must not have any contraindications to radiation.^35^

### Evaluation and Follow-Up

All patients should receive a baseline CT of the chest with or without contrast. Staging MRI of the pelvis or CT urogram is preferred before TURBT to minimize the risks of overstaging disease because of TURBT-related inflammation, which can appear similar to locally advanced MIBC. Patients who had staging after TURBT should not be excluded from clinical trials, however. An interim cystoscopy before radical cystectomy is an option, but not recommended by the SITC-IBCG panel. Given the challenging nature of clinical bladder cancer staging, this panel recommends harmonizing staging examinations and documentation in prospective trials.

Recurrence events should be confirmed by independent review. The definition of pCR to neoadjuvant therapy for MIBC should not include persistent CIS after completion of NAC. For all neoadjuvant therapy trials, patients should be followed for a minimum of 3 years with consideration of decreasing scan frequency after the first 2 years.

## MIBC TRIALS IN THE ADJUVANT SETTING

See the Data Supplement for a discussion of the following:

• Disease state definition
• Statistical and pathology considerations

### Research Hypothesis

Adjuvant systemic therapy allows treatment decisions to be based on more precise pathological rather than clinical staging. Adjuvant systemic therapy can also be used in patients with adverse pathological findings in the cystectomy specimen despite prior NAC, a situation associated with a very high risk of metastatic recurrence. A goal of perioperative systemic therapy is to eradicate micrometastases, with the rationale that systemic therapy may improve patient survival with an acceptable QOL.

### Study Objectives

The SITC-IBCG panel considers disease-free survival (DFS) and OS as the most meaningful primary end points for trials of adjuvant therapy for MIBC. In Europe, cause-specific survival (CSS) is a common primary end point; however, it is important to capture all-cause mortality including deaths due to treatment-related toxicity in the primary analysis.

Secondary end points that should be considered for adjuvant MIBC trials include both distant metastasis-free survival (DMFS, defined as recurrence outside the urothelial tract) and urothelial tract–specific RFS, patient-reported outcomes (PRO), and quality-adjusted life years (QALYs). Discontinuation rates for reasons other than recurrence should also be noted.

### Study Design

Adjuvant MIBC trials should be prospective, randomized, and controlled, generally following radical cystectomy, with allowance for treatment options following other initial management therapies such as TMT. For patients who are cisplatin-eligible and have not received NAC, cisplatin-based adjuvant therapy is considered a standard approach, although definitive recommendations are tempered by studies that were underpowered or closed early because of poor accrual. For patients who are cisplatin-ineligible or who have received prior NAC, adjuvant nivolumab is considered a treatment standard on the basis of a significant improvement in DFS.^41^

Plasma circulating tumor DNA (ctDNA) may stratify patients by micrometastatic disease presence after adjuvant treatment^42^ and should be an integral, integrated, or exploratory biomarker to inform future trial design (see the Data Supplement [Box 1] for definitions of these biomarkers, online only).

### Study Population

Eligible patients must be fit for systemic therapy and have an ECOG PS of 0-1. Adequate tumor tissue must be available for biomarker analysis. Trial populations include patients who completed SOC treatment (ie, radical cystectomy). Extent of lymph node dissection may be used as a stratification factor. Prior neoadjuvant therapy (chemotherapy or immunotherapy) should be allowed but noted with sequencing of these agents. By panel consensus and SITC definitions of resistance,^43^ prior anti–PD-(L)1 therapy should have been administered at least 6-12 months before trial inclusion if nivolumab is the comparator to avoid enrolling patients with immunotherapy-unresponsive UC onto trials of immunotherapy agents. Patients with involved surgical margins should be allowed at the discretion of the trial planning group, but if included may be capped at 20%-25% enrollment and analyzed as a subgroup. The presence of histologic subtypes >50% should be considered a stratification factor.

### Evaluation and Follow-Up

In addition to the above end points, the evaluation of clinical recurrence includes physical examination, cross-sectional imaging of the chest, abdomen, and pelvis, and consideration of other staging studies including imaging and biomarker analysis (eg, ctDNA and others^44,45^). The definition of recurrence has previously been discussed in detail.^46^ Standard radiological imaging such as CT and MRI are required, but other modalities, such as fluorodeoxyglucose (FDG) positron emission tomography (PET)/CT, could be considered as the SOC and may change in the future.^47^ The same imaging modality should be used throughout a patient's trial participation. Scans should be obtained every 3-6 months for the first 2 years, annually for the next 3 years, and as clinically indicated at 5 years and beyond. Patients should be followed for a minimum of 5 years. Distant metastasis-free survival and urothelial tract-specific RFS may be analyzed at 12 and possibly 24 months.

## METASTATIC UROTHELIAL CARCINOMA

See the Data Supplement for a discussion of the following:

• Disease state definition
• Statistical and pathology considerations
• Duration of therapy and study
• Biomarkers

### Research Hypothesis

The hypothesis for clinical trials evaluating novel agents or combinations for the treatment of metastatic UC (mUC) is that an investigational regimen will prolong survival or improve objective response rates (ORRs) compared with SOC platinum-based chemotherapy.

### Study Objectives

In patients with treatment-naïve advanced UC, primary end points should include PFS and/or OS for randomized clinical trials. Although PFS does not have good correlation with OS for trials evaluating ICIs as monotherapies,^48^ it may be considered in randomized phase II immunotherapy-based combination trials. Secondary end points should include ORR (on the basis of RECIST), durability of response, safety/toxicity, biomarkers, and QOL assessment.

For patients with progressive disease after SOC treatment, single-arm phase II trials may be conducted using RECIST v1.1 ORR for signal finding. ORR as a primary end point often excludes patients with evaluable but non-RECIST v1.1–measurable disease, particularly in patients with bone-only disease, malignant effusions, and peritoneal carcinomatosis. Alternative standard radiologic or biomarker assessment for these patients is an area of active research. Prognostic estimates to define benchmarks for estimated PFS on the basis of the specific trial population baseline characteristics may be exploratory but should be avoided as the primary end point in single-arm trials because of uncertainty, selection and confounding factors, and patient heterogeneity.

### Study Design

For randomized trials, the primary end point should be OS, defined as time from the date of random assignment to the date of death due to any cause. Patients who are still alive are censored at the last date known to be alive. OS should be estimated using the Kaplan-Meier technique.

Patients with mUC should be categorized into two groups: chemotherapy-naïve and platinum-based chemotherapy-treated (including those with completion of neoadjuvant or adjuvant chemotherapy within 12 months from the time of recurrence). The appropriate control arm in randomized first-line trials was platinum-based chemotherapy combinations (gemcitabine with carboplatin [GC], dose-dense methotrexate, vinblastine, doxorubicin, and cisplatin [ddMVAC], gemcitabine with carboplatin [GCarbo]), followed by switch maintenance avelumab at the time of manuscript publication.^49^ Enfortumab vedotin (EV) in combination with pembrolizumab is approved as a first-line treatment for cisplatin-ineligible patients, which also may be an appropriate comparator pending the results of a definitive phase III trial comparing EV plus pembrolizumab with platinum-based chemotherapy. However, the panel acknowledges that the SOC in first-line mUC is rapidly evolving and several first-line phase III trials have yet to be reported.

For patients who progress after ICI, EV is SOC for most patients and is an appropriate control arm in trials exploring therapies in patients progressing despite prior platinum-based chemotherapy and ICI in settings where EV was not previously administered. At the time of drafting this guidance, the SITC-IBCG panel considered single-agent sacituzumab govitecan or erdafitinib (in selected patients) or taxane or vinflunine as an adequate control group in randomized trials testing novel agents after progression on EV.

### Study Population

Chemotherapy-naïve patients are categorized as cisplatin-eligible versus cisplatin-ineligible. Robust randomized clinical trial data to compare cisplatin and carboplatin are not available but available data strongly suggest that cisplatin is preferred for eligible patients.^38,50^

Receipt of and time from prior platinum-based chemotherapy until the time of recurrence need to be considered. If the time from completion of prior platinum-based chemotherapy and metastatic diagnosis exceeds approximately 1 year, patients may be rechallenged. Consideration of rechallenge also depends on the pathologic response to prior NAC at the time of radical surgery, tolerance/toxicity of the regimen, PS, medical comorbidities, organ function, availability of other therapies, and clinical trials.

Patients with active autoimmune conditions requiring systemic immunosuppression should be excluded from trials evaluating ICIs. Patients with a remote (eg, >2 years) history of such conditions may be eligible depending on the type and severity of the condition.

Stratification factors in the first-line setting may include the presence of visceral metastasis and ECOG PS and/or prognostic factors/models,^51^ ideally validated in first-line trials. Albumin^52^ and neutrophil-to-lymphocyte ratio (NLR)^53^ may also be considered on the basis of sample sizes. Additionally, maintenance trials should stratify patients on the basis of response to induction platinum-based chemotherapy.

The rate of tumor growth is variable among patients with mUC. By convention, routine surveillance scans include CT of the chest, abdomen, and pelvis with IV and oral contrast, or if IV contrast is contraindicated, MRI of the abdomen and pelvis (ideally with gadolinium) and a noncontrast CT of the chest obtained at routine intervals. Initial and follow-up imaging should be the same modality. FDG-PET/CT could be a valuable adjunct^54^ but should not be used as the primary mode of response assessment. Baseline staging should be obtained within 4 weeks before starting a new therapy to ensure accurate radiologic assessment of tumor burden.

In conclusion, after decades without regulatory approval of new UC treatments, multiple novel therapies have been introduced. Optimal use of these approaches, from earlier integration to optimal treatment sequencing, requires well-designed prospective clinical trials.
